# National Surveillance of Injury in Children and Adolescents in the Republic of Korea: 2011–2017

**DOI:** 10.3390/ijerph17239132

**Published:** 2020-12-07

**Authors:** Soo Hyun Park, Ji Young Min, Won Cul Cha, Ik Joon Jo, Taerim Kim

**Affiliations:** 1 Department of Emergency Medicine, CHA Bundang Medical Center, CHA University School of Medicine, Seongnam 13496, Korea; suas11@cha.ac.kr; 2 Department of Digital Health, Samsung Advanced Institute for Health Science & Technology (SAIHST), Sungkyunkwan University, Seoul 06355, Korea; wnet50094@gmail.com; 3 Department of Emergency Medicine, Samsung Medical Center, Sungkyunkwan University School of Medicine, Seoul 06351, Korea; wc.cha@samsung.com (W.C.C.); ikjoon.jo@samsung.com (I.J.J.)

**Keywords:** wounds and injuries, epidemiology, pediatrics, prevention and control

## Abstract

Understanding age-specific injury patterns allows the continued improvement of prevention strategies. This is a retrospective study analyzing the Korea Emergency Department-Based Injury In-depth Surveillance data, including those aged ≤19 years old between January 2011 and December 2017. In this study, we focused on changes in the modes of injury and severity, and prevention potential by dividing the patients into four age groups: group 1 (0–4 years), group 2 (5–9 years), group 3 (10–14 years), and group 4 (15–19 years). The most common mode of injury in younger age groups 1 and 2 was a fall or slip. Most injuries in older age groups 3 and 4 were unintentional and intentional collisions combined. Traumatic brain injuries (2.1%), intensive care unit admissions (1.8%), and overall death (0.4%) were the highest in group 4. The proportions of severe and critical injury (EMR-ISS ≥ 25) were 7.5% in group 4, 3.2% in group 3, 2.5% in group 1, and 1% in group 2. This study presents a comprehensive trend of injuries in the pediatric population in South Korea. Our results suggest the importance of designing specific injury-prevention strategies for targeted groups, circumstances, and situations.

## 1. Introduction

Injuries are a leading cause of morbidity and mortality, especially in children and adolescents worldwide. In 2016, the World Health Organization (WHO) reported that over 644,855 children under the age of 15 died due to injury, and there were 10–30 million non-fatal injuries. Mortality is the most important indicator of injuries as a health problem. However, injuries that do not lead to death cannot be overlooked because these result in hospitalization, crowded emergency departments (EDs), treatment by general physicians or other health personnel, and use of medical resources [1]. Injuries require acute and rehabilitative care.

According to the National Injury Fact Book of the Korea Centers for Disease Control and Prevention (KCDC) in 2016, 15,769 injuries were reported among children aged 0–9 years per 100,000 people, which was the highest proportion of the total incidence of injuries [2]. The Korea Statistical Office (KOSTAT) reported that 14.8% of deaths among children aged 0–14 years in Korea in 2016 were due to injury [3]. In 2015, South Korea ranked ninth in injury-related death in children among 32 Organization for Economic Cooperation and Development (OECD) countries [3]. Child mortality from unintentional injury in South Korea needs to be reduced.

In the past, injuries were viewed as “accidents” or “trauma,” resulting in the need for prevention being neglected [1]. Since the late 1940s, epidemiological analyses of injuries have been used to plan prevention strategies, and the view of injuries has changed to preventable events [4,5]. Several prevention plans around the world have been carried out to prevent pediatric injury. The WHO has documented such child and adolescent injury prevention plans: WHO Plan of Action (2006–2015), World Report on Violence and Health, and World Report on Child Injury Prevention [6]. In addition, there are the ‘INSPIRE’ Seven Strategies for Ending Violence Against Children [7], the U.S.A.’s National Action Plan for Child Injury Prevention [8], and the European Report on Child Injury Prevention [9]. Regardless, injuries continue to be the cause of morbidity and mortality in the pediatric population in the 21st century [10].

The development and growth of children differ markedly with age [11,12]. Therefore, there is a big difference in the mechanism of injury according to age. Several studies have shown differences in the mechanisms of injury by age [13,14,15]. Times and cultures are rapidly changing, so it is important to keep track of epidemiological trends of injury in children. Tracking these trends will help to prevent injury.

It is important to understand the various injury patterns and modes to establish a national plan for prevention over the long term. Epidemiological studies on injuries in the pediatric population are the bases of prevention [5,16]. Research on pediatric injury has been growing steadily [16]. This study aimed to investigate the epidemiologic characteristics of both unintentional and intentional injuries in the pediatric population presenting to the EDs and to recommend measures for injury-preventive plans.

## 2. Materials and Methods

### 2.1. Study Design

This study retrospectively analyzed data from the Emergency Department-based Injury In-depth Surveillance (EDIIS) database in South Korea. Data were obtained from the electronic medical records written by physicians. Then they were collected and reviewed from the standardized registry by the trained coordinator. The data quality was monitored by the KCDC regularly. EDIIS is the largest high-quality injury database from Emergency Departments in Korea. The EDIIS database is based on the dataset of the International Classification of External Causes of Injuries (ICECI) maintained by the WHO. We used a database from the population statistics of Ministry of the Interior and safety on the total pediatric population over time to compare with the number of pediatric injuries [17].

The study population included children and adolescents aged ≤19 years old who had sustained injuries and visited EDs between January 2011 and December 2017. Age classifications from the Centers for Disease Control and Prevention in the U.S. are: younger than 1 year; 1 to 4 years; 5 to 9 years; and 10 to 14 years [18]. In our study, we grouped under 1 and 1 to 4-year-olds into group 1 (0–4), and the remaining into group 2 (5–9 years), group 3 (10–14 years), and group 4 (15–19 years).

The study was approved by the Institutional Review Board of Samsung Seoul Hospital (IRB file number 2020-02-030) and the KCDC. This study was exempt from the requirement of informed consent owing to the retrospective design.

### 2.2. Measures

The demographic factors included sex and age. The prehospital factors were emergency mode of arrival, place, kind of activity, and time. Times of injury were classified as day (07:00 to 14:59), evening (15:00 to 22:59), and night (23:00 to 6:59) according to the 8-h hospital shift [19], and days of injury were divided as Monday to Friday, as weekdays, and Saturday to Sunday, as the weekend. 

To identify the severity and mortality, we added variables indicating poor clinical course, such as discharge from ED (admission to general ward or intensive care unit (ICU), transfer, or death), operation, EMR-ISS, overall death, and traumatic brain injury (TBI). For admitted patients, length of stay and death in the hospital were also analyzed.

The mechanism of injury in EDIIS data from KCDC was modified on the basis of the International Classification of External Causes of Injury (ICECI) from the WHO (Table S1). Among the injury mechanisms, we included fall or slip, collision (stuck), penetration (cut/pierce), overuse (overexertion), thermal injury (burn), motor vehicle, substance exposure (poisoning and adverse effect), drowning, hanging, asphyxia (suffocation), machine and natural disasters. In addition, the remaining few mechanisms were combined and classified into others and unknown.

Injury severity was determined on the basis of the excess mortality ratio-adjusted injury severity scale (EMR-ISS) [20]. This scale is divided into four stages: 1 ≤ EMR-ISS ≤ 8 (mild), 9 ≤ EMR-ISS ≤ 24 (moderate), 25 ≤ EMR-ISS ≤ 74 (severe), and EMR-ISS ≥ 75 or death (critical) [20]. TBI was one of the important clinical outcomes that was defined by International Classification of Diseases, 10th Revision (ICD-10) codes: F07.2, S02.0, S02.1, S02.3, S02.7, S02.8, S02.9, S06, S07.1, T90.2, and T90.5.

The injured anatomical sites were categorized as head (S00–S09), neck (S10–S19), thorax (S20–29), abdomen and pelvis including lower back and genitals (S30–S39), shoulders and upper arms (S40–S49), elbows and lower arm (S50–S59), wrists and hands (S60–S69), hips and thighs (S70–S79), knees and lower legs (S80–S89), and ankle and feet (S90–S99), according to the ICD-10.

Data on protective gears have been collected since 2016. Data on safety seat belts and air bag installations and inflation were collected in those aged 6–19 years old and presence of a car safety seat was checked for those below 6 years old only for in-car traffic events. Adolescents in motorcycle events between ages 10 and 19 years were checked for the presence of a helmet. All pediatric age groups in bicycle-related events were checked for the presence of a helmet.

### 2.3. Statistical Analysis

Continuous variables were described as medians with interquartile ranges (IQRs), and categorical variables were described as frequencies with percentages. The Kruskal–Wallis test for continuous values and the Chi-squared test for categorical values were used for comparisons among age groups. *p* < 0.05 was considered to indicate statistical significance in all statistical tests. R statistical software version 3.6.0 (R Foundation for Statistical Computing, Vienna, Austria) as used for statistical analysis. 

## 3. Results

### 3.1. General Features of Pediatric Injury

**Figure 1:**
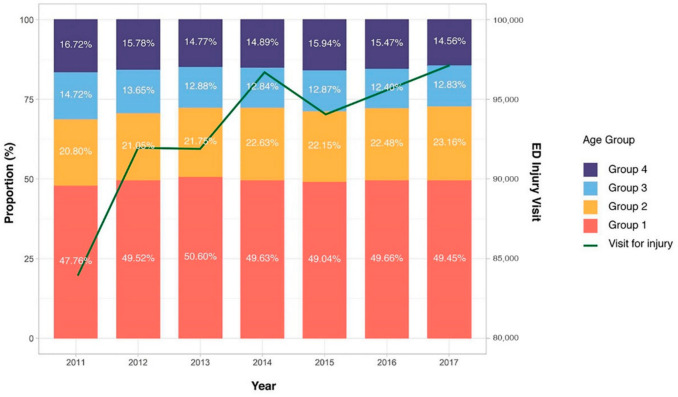
Proportion of emergency department (ED) visits for injury by age group during 2011–2017.

In South Korea, there were 1,830,904 hospital visits related to injury during 2011–2017. We excluded visiting patients older than 20 years (*n =* 1,179,772); 651,132 ED visits for injuries in children were eligible for inclusion (Figure S1). Figure S2 depicts the entire pediatric population by year in South Korea. This helps to understand the pediatric injury pattern. The number of ED visits by year increased in a bimodal pattern (Figure 1 and Table S2). Age groups were categorized as follows: group 1 (*n =* 321,671), group 2 (*n =* 143,475), group 3 (*n =* 85,574), and group 4 (*n =* 100,412). Group 1 had the highest ED visits, accounting for 49.4% of the total. 

Group 1 has the highest proportion of visits in all years. The injury counts showed a bimodal pattern, with peaks in 2014 and 2017.

### 3.2. Demographics of Pediatric Injuries in the ED

The demographic and prehospital information is summarized in Table 1. The proportion of male patients was 72.1% in group 3, 70.0% in group 4, 63.7% in group 2, and 59.1% in group 1. Use of emergency medical services (119) was higher in the older age groups (group 4, 21.2%; group 3, 12.1%; group 2, 7.5%; and group 1, 5.9%). In younger age groups 1 and 2, the most common place of injury was the house (group 1, 74.8%; group 2, 44.6%). Furthermore, the rates of injury on the road (group 4, 33.4%; group 3, 23.0% vs. group 2, 17.6%; group 1, 8.0%) and school and educational facilities (group 3, 21.9%; group 4, 13.7% vs. group 2, 10.1%; group 1, 3.5%) were higher in the adolescents. The most common activities that led to injury were activities of daily living in all groups.

However, in the older age groups, the frequency of injuries caused by other activities increased. Furthermore, 95.4% and 91.3% in groups 1 and 2, respectively, used national health insurance, while 13.7% in group 4 used vehicle insurance. Regarding time of injury, evening was the most common time for all age groups. However, nighttime (25.0%) showed an increase in group 4. Injury was more common on weekdays in all age groups.

### 3.3. Mechanisms of Pediatric Injuries

**Figure 2:**
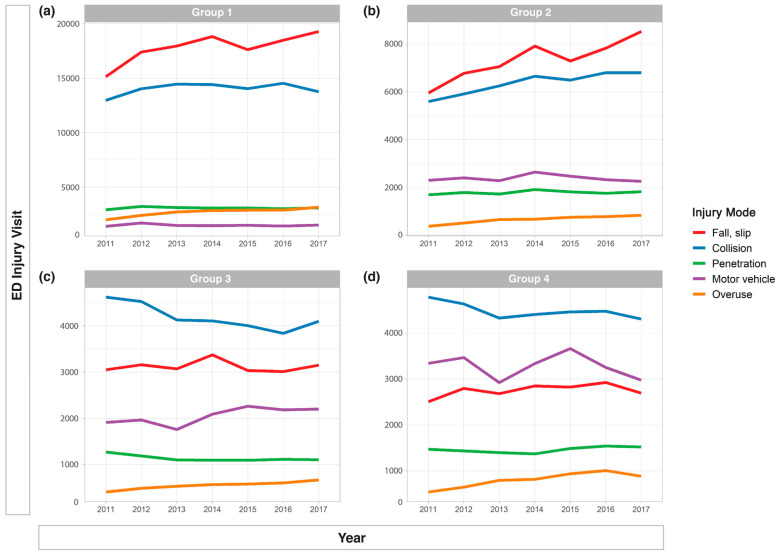
Five most common modes of injury by age group. (**a**) group 1 (0 to 4 years), (**b**) group 2 (5 to 9 years), (**c**) group 3 (10 to 14 years), (**d**) group 4 (15 to 19 years).

**Figure 3:**
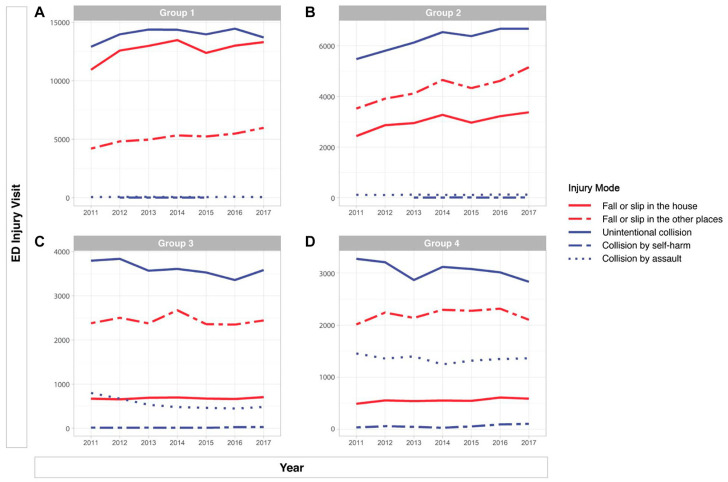
Specific analysis graph of place of injury vs. intention. (**a**) group 1 (0 to 4 years), (**b**) group 2 (5 to 9 years), (**c**) group 3 (10 to 14 years), (**d**) group 4 (15 to 19 years).

Figure 2 presents the five most common modes of injury by age group. In group 1, injuries were most commonly caused by a fall or slip (38.8%), collision (30.5%), penetration (6.7%), overuse (5.9%), and thermal causes (3.9%); in group 2, by fall or slip (35.8%), collision (31.0%), motor vehicle (11.6%), penetration (8.7%), and overuse (3.1%); in group 3, by collision (34.3%), fall or slip (25.5%), motor vehicle (16.8%), penetration (9.3%), and overuse (4.5%); and in group 4, by collision (31.3%), motor vehicle (22.8%), fall or slip (19.2%), penetration (10.2%), and overuse (5.6%). The most common mode of injury in the younger age groups (groups 1 and 2) was fall or slip. In group 1, the occurrence of fall or slip was significantly higher in the house. In group 2, it occurred in other places apart from the house (Figure 3A,B). Most common cause of injuries in older age groups (group 3 and 4) was collision. In collision cases, unintentional injuries were common in both groups. However, the rate of assault was higher in group 4 than in group 3 (Figure 3C,D). Table 1 shows that intentional injuries increased in older groups.

### 3.4. Anatomical Site of Injury

**Figure 4:**
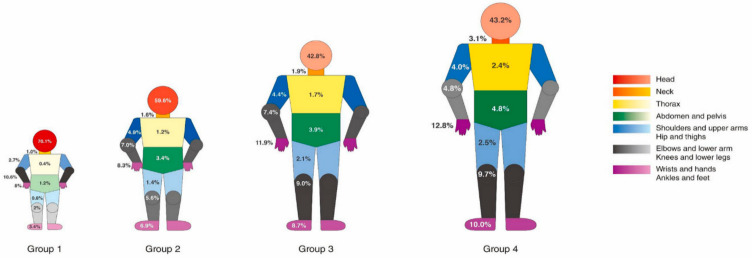
Anatomical sites of the injury can be seen according to age group. The sites that are more prone to injury are shaded with a darker color. Group 1 (0 to 4 years), group 2 (5 to 9 years), group 3 (10 to 14 years), group 4 (15 to 19 years).

The most common anatomical site of injury was the head (group 1, 70.1%; group 2, 59.6%; group 3, 42.8%; group 4, 43.2%) in all age groups. In the younger age group (groups 1 and 2), the rate of head injuries was high. However, injuries to the ankles and feet were more common in group 4 (10.0%) and group 3 (8.7%) than in group 2 (6.9%) and group 1 (3.4%) (Figure 4).

### 3.5. Clinical Results of Pediatric Injury Patients by Age Group

The overall clinical results are presented in Table 2. In all, 96.9% (group 1), 93.7% (group 2), 90.7% (group 3), and 86.1% (group 4) of the patients were discharged from the EDs. The proportions of discharge against medical advice were 1.9% for group 4, 0.6% for group 3, 0.4% for group 2, and 0.4% for group 1. The ward admission rates were 7.7% for group 4, 6.7% for group 3, 4.8% for group 2, and 2.0% for group 1. TBI (2.1%), ICU care (1.8%), and overall death (0.4%) were highest in group 4. The proportions of severe and critical injury (EMR-ISS ≥ 25) were 7.5% (group 4), 3.2% (group 3), 2.5% (group 1), and 2.1% (group 2). The median value (IQR) of hospital stay for inpatients was 7.6 (3.7–14.8) days for group 4, 4.8 (2.7–8.8) days for group 3, 3.7 (2.0–7.0) days for group 2, and 2.8 (1.7–5.7) days for group 1 (Table S3).

### 3.6. Protective Gear Use

The proportions of motor vehicle injury cases who had used safety seat belts were 45.1%, 43.7%, and 42.3% in the 10–14 year, 6–9 year, and 15–19-year age groups, respectively. The safety seat belts were worn in all age groups in fewer than half of all cases (Table S4). The proportions of in-car traffic injury cases who had used car safety seats were 46.3% in the 0–1-year-old age group, 38.4% in the 2–3-year-old age group, and 28.5% in the 4–5-year-old age group (Table S5). Adolescents wore a helmet in motorcycle injuries in 34.2% of the cases in the 15–19-year-old age group and 13.0% of the cases in the 10–14-year-old age group (Table S6). Fewer than 10% of the children in all age groups in bicycle-related injuries wore helmets (group 4, 9.0%; group 2, 8.5%; group 3, 8.5%; group 1, 7.8%) (Table S7).

## 4. Discussion

Our study has several strengths. Firstly, it was a nationwide multicenter study with data across eight years. In the study conducted by Jung et al. [21], the epidemiology of pediatric injury was presented only for unintentional injury over two years (2010–2011). Two years is a short period to see the trends in overall pediatric injury. Therefore, our study showed age-specific injury patterns. The environments of pediatric injury varied by age groups. Secondly, we analyzed both unintentional and intentional injuries including prehospital factors and modes to show the trends in a broader sense. Several papers have investigated the epidemiology of pediatric injury by analyzing specific places or specific modes [22,23,24]. However, this is not enough to establish national preventive measures. The epidemiology of injuries in the entire pediatric population is an important indicator for national health plans and distribution of medical resources.

In our study, the most common mode of injury in the younger age groups was a fall or slip. However, the difference between groups 1 and 2 was the location of injury. Studies have reported younger children slipping from the caregiver’s arms or being inappropriately placed on furniture [25,26]. Therefore, parents/caregivers should be careful when using a baby carrier, avoid hazards, and supervise younger children in bed. Similarly, Unni et al. [25] showed that fall or slip injuries occurred in places other than the house in group 2. Injury prevention efforts should target not only parents but also kindergarten or elementary school teachers because these injuries can be related to education and sport facilities. Preventive interventions should involve age-specific plans and population-based targeted education for parents or caregivers. In addition, physicians in EDs can determine age-related injuries and give age-appropriate recommendations to caregivers.

Figure 3C,D presents the differences in the types of collisions between group 3 and group 4. In particular, a high rate of assaults is shown in group 4. We could not identify the perpetrator from the data, but mode of assault included both child abuse and peer-related violence. In some studies, ED visits for assault were usually observed in the low-income class [27]. This population is small and socially vulnerable; hence, it is necessary to pay proper attention to them in EDs. ED-based screening is one method for the detection of child abuse. Several studies have reported successful results with case management interventions for peer-related violence [28,29]. Interventions including peer education or social programs should be necessary not only for group 4 but also for group 3, as the proportion of assaults showed an increase in this group.

In our data, visits for self-harm injury represented a small subset. However, injury caused by self-harm increased in group 4 (3.6%) vs. group 3 (0.7%). Self-harm in adolescents is a major public health concern [30]. Self-harm injuries need specific care and are associated with substantial resources and financial burden on EDs [27]. Some studies recommend interventions such as screening or staff training in crisis intervention [31,32]. Further studies are needed to improve interventions for pediatric populations at a high risk of suicide. Furthermore, national guidelines on the prevention of self-harm and suicide are needed.

Regardless of age, the most common site of injury was the head. The youngest group, group 1, had the highest rate of head injury (70.1%) associated with falls [25]; hence, physicians should consider the possibility of falls in the EDs. As the age increases, the modes vary, resulting in injury to various body sites. Injury to wrists and hands (12.8%) or ankles and feet (10.0%) was higher in group 4 than in the other groups. These results should be considered by physicians in the EDs and by policymakers for designing safe facilities for children.

Tables S3 and S4 show that protective equipment is not worn in all age groups. It is very important to ensure that the pediatric population uses proper safety restraint systems in vehicles, such as seat belts, and boosters and car safety seats for infant and child passengers, because they reduce the risk of serious injury [33,34]. However, our results show that fewer than half of the cases used a seat belt and a car safety seat. Parental education is an important factor in the use of proper safety restraints. Education campaigns can significantly increase the proper use of safety restraints [35,36]. The rate of use of child safety restraint systems is significantly lower in South Korea than in advanced countries, where the rate exceeds 90%. In September 2018, legislation mandating the use of car seat belts in the back seats and car safety seats for children aged below six years was imposed in South Korea [37]. However, the use of safety restraints has not increased. In some developed countries such as Canada, Japan, and several states in the United States, penalties are imposed on drivers who do not keep the laws on safety restraint. It is necessary to ensure compliance and adherence to legislation and provision of legal remedies.

Another important piece of protective gear is the helmet. In our study, fewer than 10% of the children in all age groups wore a helmet in bicycle-related injuries. The rate of helmet use in motorcycle-related injuries was also significantly low. TBI often occurs due to motorcycle and bicycle events in the pediatric population [38]. Some studies support the efficacy of mandatory helmet use legislation in decreasing head injuries and mortality among children and adolescents [39,40]. In South Korea, the Road Traffic legislation was revised to make it mandatory to wear a helmet as of September 2018 [37]. However, there is no actual enforcement or punishment for not obeying these guidelines. Regulation of legislations on wearing the helmet and safety initiatives is needed to prevent TBI due to motorcycles and bicycles.

### Limitations

Firstly, this retrospective study used data obtained from the EDIIS. The data may be subject to individual coding variations and code error, but because the database has nationwide data, this limitation could be overcome. Secondly, this study included only patients presenting to EDs, and this is likely to cause selection bias because patients who are already dead or have minor injury and do not need treatment may not visit the EDs. Thirdly, there is the difference in the number of centers by year. There were 20 participating EDs during 2011–2014, but 23 EDs during 2015–2017. Additionally, we used the EMR-ISS instead of the International Classification of Diseases (ICD)-9 Based Injury Severity Score (ICISS). The EMR-ISS was adapted from the International Statistical Classification of Diseases and Related Health Problems, 10th Revision, Clinical Modification (ICD-10-CM) [20]. The use of the EMR-ISS in other countries has not yet been reported, but multiple studies in Korea have used it.

## 5. Conclusions

Our analysis presents a comprehensive trend of injury in children and adolescents in South Korea. Our study strongly recommends developing specific injury-prevention strategies for targeted groups, circumstances, and situations through safety education, enforcement of legislations, and public campaigns.

## Figures and Tables

**Table 1:** General characteristics of pediatric injury patients by age group.

Characteristic	No. (%)				*p*-Value
	Group 1 (0–4) (*n* = 321,671)	Group 2 (5–9) (*n* = 143,475)	Group 3 (10–14) (*n* = 85,574)	Group 4 (15–19) (*n* = 100,412)	
**Sex**					<0.0001
- Male	190,172 (59.1%)	91,344 (63.7%)	61,731 (72.1%)	70,296 (70.0%)	
- Female	131,499 (40.9%)	52,131 (36.3%)	23,843 (27.9%)	30,116 (30.0%)	
**Mode of Arrival**					<0.001
- Walk-in	298,448 (92.8%)	130,220 (90.8%)	72,998 (85.3%)	75,114 (74.8%)	
- EMS (119)	19,135 (5.9%)	10,690 (7.5%)	10,326 (12.1%)	21,263 (21.2%)	
- Private ambulance	2560 (0.8%)	2283 (1.6%)	2074 (2.4%)	3690 (3.7%)	
- Others and unknown	1528 (0.5%)	282 (0.2%)	176 (0.2%)	345 (0.3%)	
**Place**					<0.001
- House	240,554 (74.8%)	63,932 (44.6%)	23,616 (27.6%)	23,472 (23.4%)	
- Commercial facilities and amusement, public facilities	30,242 (9.4%)	22,183 (15.5%)	8690 (10.2%)	12,861 (12.8%)	
- Road	25,787 (8.0%)	25,236 (17.6%)	19,720 (23.0%)	33,586 (33.4%)	
- School, education facilities	11,140 (3.5%)	14,560 (10.1%)	18,744 (21.9%)	13,740 (13.7%)	
- Outdoor, river, sea	5542 (1.7%)	5677 (4.0%)	2829 (3.3%)	2927 (2.9%)	
- Sport facilities	1935 (0.6%)	8208 (5.7%)	9953 (11.6%)	9924 (9.9%)	
- Residential facilities	1930 (0.6%)	1235 (0.9%)	591 (0.7%)	1232 (1.2%)	
- Medical facilities	1420 (0.4%)	373 (0.3%)	179 (0.2%)	294 (0.3%)	
- Farm, factory, industrial facilities	291 (0.1%)	266 (0.2%)	160 (0.2%)	874 (0.9%)	
- Others and unknown	2830 (0.9%)	1805 (1.3%)	1092 (1.3%)	1502 (1.5%)	
**Activity**					<0.001
- Daily living activities	254,615 (80.4%)	77,781 (55.6%)	31,381 (39.6%)	33,297 (41.3%)	
- Leisure activities	54,193 (17.1%)	45,384 (32.5%)	23,739 (29.9%)	23,413 (29.0%)	
- Education	6563 (2.1%)	10197 (7.3%)	12,982 (16.4%)	9027 (11.2%)	
- Exercise	622 (0.2%)	5665 (4.1%)	9918 (12.5%)	10,914 (13.5%)	
- Others and unknown	885 (0.3%)	753 (0.5%)	1259 (1.6%)	4002 (5.0%)	
**Insurance**					<0.001
- National Health Insurance	307,029 (95.4%)	131,044 (91.3%)	74,774 (87.4%)	77,745 (77.4%)	
- Vehicle	6490 (2.0%)	8461 (5.9%)	6736 (7.9%)	13,798 (13.7%)	
- Self-pay (uninsured)	3708 (1.2%)	1499 (1.0%)	1418 (1.7%)	4128 (4.1%)	
- Medicaid beneficiary	3321 (1.0%)	2133 (1.5%)	2443 (2.9%)	4377 (4.4%)	
- Others and unknown	1123 (0.3%)	338 (0.2%)	203 (0.2%)	364 (0.4%)	
**Day of injury**					<0.001
- Weekday (Monday–Friday)	194,227 (60.4%)	85,475 (59.6%)	56,284 (65.8%)	65,917 (65.6%)	
- Weekend (Saturday–Sunday)	127,408 (39.6%)	57,984 (40.4%)	29,280 (34.2%)	34,489 (34.3%)	
**Time of injury**					<0.001
- Day (07:00–14:59)	93,061 (28.9%)	42,596 (29.7%)	31,304 (36.6%)	26,089 (26.0%)	
- Evening (15:00–22:59)	202,946 (63.1%)	92,837 (64.7%)	48,334 (56.5%)	48,996 (48.8%)	
- Night (23:00–06:59)	25,198 (7.8%)	7848 (5.5%)	5810 (6.8%)	25,145 (25.0%)	
**Day of ED visit**					<0.001
- Weekday (Monday–Friday)	194,644 (60.5%)	85,601 (59.7%)	55,991 (65.4%)	65,390 (65.1%)	
- Weekend (Saturday–Sunday)	127,027 (39.5%)	57,874 (40.3%)	29,583 (34.6%)	35,022 (34.9%)	
**Time of ED visit**					<0.001
- Day (07:00–14:59)	83,848 (26.1%)	34,913 (24.3%)	23,794 (27.8%)	23,819 (23.7%)	
- Evening (15:00–22:59)	207,015 (64.4%)	99,410 (69.3%)	54,292 (63.4%)	49,754 (49.5%)	
- Night (23:00–06:59)	30,807 (9.6%)	9152 (6.4%)	7488 (8.8%)	26,839 (26.7%)	
**Mode**					<0.001
- Fall, slip	124,694 (38.8%)	51,386 (35.8%)	21,834 (25.5%)	19,258 (19.2%)	
- Collision	98,207 (30.5%)	44,525 (31.0%)	29,317 (34.3%)	31,389 (31.3%)	
- Penetration	21,567 (6.7%)	12,459 (8.7%)	7962 (9.3%)	10,203 (10.2%)	
- Overuse	18,924 (5.9%)	4518 (3.1%)	3842 (4.5%)	5602 (5.6%)	
- Thermal injury	12,531 (3.9%)	2635 (1.8%)	1577 (1.8%)	2182 (2.2%)	
- Motor vehicle	10,511 (3.3%)	16,641 (11.6%)	14,369 (16.8%)	22,942 (22.8%)	
- Substance exposure	4557 (1.4%)	579 (0.4%)	833 (1.0%)	2608 (2.6%)	
- Drowning, hanging, asphyxia	456 (0.1%)	154 (0.1%)	131 (0.2%)	207 (0.2%)	
- Machine	131 (0.0%)	45 (0.0%)	28 (0.0%)	228 (0.2%)	
- Natural disaster	1 (0.0%)	1 (0.0%)	0 (0.0%)	5 (0.0%)	
- Others and unknown	30,092 (9.4%)	10,532 (7.3%)	5681 (6.6%)	5788 (5.8%)	
**Intention**					<0.001
- Unintentional	320,876 (99.8%)	142,469 (99.3%)	80,782 (94.4%)	86,538 (86.2%)	
- Assault	440 (0.1%)	868 (0.6%)	4023 (4.7%)	9849 (9.8%)	
- Self-harm, suicide	21 (0.0%)	17 (0.0%)	611 (0.7%)	3628 (3.6%)	
- Others and unknown	334 (0.1%)	121 (0.1%)	158 (0.2%)	397 (0.4%)	

EMS, emergency medical services; ED, emergency department.

**Table 2:** Outcomes of pediatric injury patients by age group.

Outcome	No. (%)				*p*-Value
	Group 1 (0–4) (*n =* 321,671)	Group 2 (5–9) (*n =* 143,475)	Group 3 (10–14) (*n =* 85,574)	Group 4 (15–19) (*n =* 100,412)	
**ED dis** **position**					<0.001
- Discharge	311,619 (96.9%)	134,418 (93.7%)	77,654 (90.7%)	86,434 (86.1%)	
- Admission to general ward	6501 (2.0%)	6862 (4.8%)	5761 (6.7%)	7755 (7.7%)	
- Against medical advice	1375 (0.4%)	515 (0.4%)	551 (0.6%)	1907 (1.9%)	
- Transfer	1211 (0.4%)	894 (0.6%)	857 (1.0%)	2081 (2.1%)	
- ICU	729 (0.2%)	660 (0.5%)	666 (0.8%)	1841 (1.8%)	
- Death in ED	95 (0.0%)	68 (0.0%)	55 (0.1%)	296 (0.3%)	
- Others and unknown	141 (0.0%)	58 (0.0%)	30 (0.0%)	98 (0.1%)	
**Operation**					<0.001
- No	208,767 (64.9%)	92,066 (64.2%)	53,123 (62.1%)	62,320 (62.1%)	
- Yes	3544 (1.1%)	3621 (2.5%)	2768 (3.2%)	3652 (3.6%)	
- Unknown	109,360 (34.0%)	47,788 (33.3%)	29,683 (34.7%)	34,440 (34.3%)	
**EMR-ISS**					<0.001
- Mild (1 ≤ EMR-ISS < 9)	124,452 (38.7%)	58,917 (41.1%)	43,570 (50.9%)	49,262 (49.1%)	
- Moderate (9 ≤ EMR-ISS < 25)	186,670 (58.0%)	80,281 (56.0%)	38,656 (45.2%)	42,832 (42.7%)	
- Severe (25 ≤ EMR-ISS < 75)	7756 (2.4%)	2833 (2.0%)	2577 (3.0%)	6801 (6.8%)	
- Critical (EMR-ISS = 75 or death)	234 (0.1%)	193 (0.1%)	162 (0.2%)	663 (0.7%)	
**ED stay time, median (IQR), hours**	1.4 (0.7–2.6)	1.5 (0.9–2.6)	1.6 (0.9–2.7)	1.8 (1.0–3.3)	<0.001
**Overall death**					<0.001
- No	321,501 (99.9%)	143,357 (99.9%)	85,475 (99.9%)	99,961 (99.6%)	
- Yes	170 (0.1%)	118 (0.1%)	99 (0.1%)	451 (0.4%)	
**Traumatic brain injury (TBI)**					<0.001
- No	319,650 (99.4%)	142,725 (99.5%)	84,646 (98.9%)	98,328 (97.9%)	
- Yes	2021 (0.6%)	750 (0.5%)	928 (1.1%)	2084 (2.1%)	

EMR-ISS, excess mortality ratio-adjusted injury severity scale.

## Supplementary Materials

The following are available online at https://www.mdpi.com/1660-4601/17/23/9132/s1, Figure S1. Flowchart of patient enrollment. Figure S2. Emergency department visits for injury of the entire pediatric population (bars) (graph). Table S1. ICECI version 1.2 and EDIIS code equivalents: Mechanism/cause. Table S2. Emergency department visits for injury by pediatric age group and year. Table S3. Hospital length of stay and death of pediatric injury inpatients. Table S4. (A) The number of children aged ≥6 years who wore a safety seat belt and experienced air bag installation at the time of the car crash. (B) The number of children aged 6 years who experienced air bag inflation at the time of the car crash. Table S5. The Number of children aged under 6 years sitting on the car safety seat at the time of the car crash. Table S6. The number of adolescents wearing helmet, aged ≥10 years, at the time of motorcycle-related injury as a driver or a passenger. Table S7. The number of individuals wearing helmet by age group at the time of the bicycle-related injury.

## References

[1] Krug E.G., Sharma G.K., Lozano R. (2000). The global burden of injuries. Am. J. Public Health.

[2] Korea Centers for Disease Control and Prevention (KCDC) (2018). National Injury Fact Book 2015–2016.

[3] Korea Statistical Office (KOSTAT) Child Death Due to Injury (1996–2016). http://kostat.go.kr/portal/korea/kor_nw/1/6/1/index.board?bmode=read&bSeq=&aSeq=367573&pageNo=3&rowNum=10&navCount=10&currPg=&searchInfo=&sTarget=title&sTxt=.

[4] Gordon J.E. (1949). The epidemiology of accidents. Am. J. Public Health Nat. Health.

[5] Haddon W. (1968). The changing approach to the epidemiology, prevention, and amelioration of trauma: The transition to approaches etiologically rather than descriptively based. Am. J. Public Health Nat. Health.

[6] Peden M., Oyegbite K., Ozanne-Smith J., Hyder A.A., Branche C., Rahman A.F., Rivara F., Bartolomeos K. (2008). World Report on Child Injury Prevention.

[7] World Health Organization (2016). INSPIRE: Seven Strategies for Ending Violence against Children.

[8] Baldwin G., Sleet D., Gilchrist J., Degutis L. (2012). Fulfilling a promise: The national action plan for child injury prevention. Inj. Prev..

[9] Sethi D. (2008). European Report on Child Injury Prevention.

[10] Dorney K., Dodington J.M., Rees C.A., Farrell C.A., Hanson H.R., Lyons T.W., Lee L.K. (2020). The Injury Free Coalition for Kids. Preventing injuries must be a priority to prevent disease in the twenty-first century. Pediatr. Res..

[11] Flavin M.P., Dostaler S.M., Simpson K., Brison R.J., Pickett W. (2006). Stages of development and injury patterns in the early years: A population-based analysis. BMC Public Health.

[12] Figaji A.A. (2017). Anatomical and Physiological Differences between Children and Adults Relevant to Traumatic Brain Injury and the Implications for Clinical Assessment and Care. Front. Neurol..

[13] Tracy E.T., Englum B.R., Barbas A.S., Foley C., Rice H.E., Shapiro M.L. (2013). Pediatric injury patterns by year of age. J. Pediatr. Surg..

[14] Aoki M., Abe T., Saitoh D., Oshima K. (2019). Epidemiology, Patterns of treatment, and Mortality of Pediatric Trauma Patients in Japan. Sci. Rep..

[15] Sengoelge M., Hasselberg M., Laflamme L. (2011). Child home injury mortality in Europe: A 16-country analysis. Eur. J. Public Health.

[16] Sleet D.A. (2018). The Global Challenge of Child Injury Prevention. Int. J. Environ. Res. Public Health.

[17] Korea Ministry of the Interior and Safety Total Population Statistics. http://27.101.213.4/.

[18] Borse N., Sleet D.A. (2009). CDC Childhood Injury Report: Patterns of Unintentional Injuries Among 0- to 19-Year Olds in the United States, 2000–2006. Fam. Community Health.

[19] Rathlev N.K., Obendorfer D., White L.F., Rebholz C., Magauran B., Baker W., Ulrich A., Fisher L., Olshaker J. (2012). Time series analysis of emergency department length of stay per 8-hour shift. West. J. Emerg. Med..

[20] Kim J., Shin S.D., Im T.H., Kug Jong L., Ko S.B., Park J.O., Ahn K.O., Song K.J. (2009). Development and validation of the Excess Mortality Ratio-adjusted Injury Severity Score Using the International Classification of Diseases 10th Edition. Acad. Emerg. Med..

[21] Jung J.H., Kim D.K., Jang H.Y., Kwak Y.H. (2015). Epidemiology and Regional Distribution of Pediatric Unintentional Emergency Injury in Korea from 2010 to 2011. J. Korean Med. Sci..

[22] Choi I.C., Park J.W., Jung J.Y., Kim D.K., Kwak Y.H., Suh D., Lee S.U. (2020). Pediatric Injuries in Kids Cafes and Risk Factors for Significant Injuries: A 6-Year Cross-Sectional Study Using a Multicenter Injury Registry in Korea. J. Korean Med. Sci..

[23] Cook J.A., Sasor S.E., Soleimani T., Chu M.W., Tholpady S.S. (2020). An Epidemiological Analysis of Pediatric Dog Bite Injuries Over a Decade. J. Surg. Res..

[24] Hanson H.R., Gittelman M.A., Pomerantz W.J. (2019). Trends of ED visits, admissions, and deaths for pediatric traumatic brain injury comparing sport and non-sport mechanisms. Inj. Epidemiol..

[25] Unni P., Locklair M.R., Morrow S.E., Estrada C. (2012). Age variability in pediatric injuries from falls. Am. J. Emerg. Med..

[26] Chaudhary S., Figueroa J., Shaikh S., Mays E.W., Bayakly R., Javed M., Smith M.L., Moran T.P., Rupp J., Nieb S. (2018). Pediatric falls ages 0–4: Understanding demographics, mechanisms, and injury severities. Inj. Epidemiol..

[27] Ballard E.D., Kalb L.G., Vasa R.A., Goldstein M., Wilcox H.C. (2015). Self-harm, Assault, and Undetermined Intent Injuries Among Pediatric Emergency Department Visits. Pediatr. Emerg. Care.

[28] Snider C., Lee J. (2009). Youth violence secondary prevention initiatives in emergency departments: A systematic review. CJEM.

[29] Cheng T.L., Wright J.L., Markakis D., Copeland-Linder N., Menvielle E. (2008). Randomized trial of a case management program for assault-injured youth: Impact on service utilization and risk for reinjury. Pediatr. Emerg. Care.

[30] Hawton K., Saunders K.E., O’Connor R.C. (2012). Self-harm and suicide in adolescents. Lancet.

[31] Gould M.S., Munfakh J.L., Kleinman M., Lake A.M. (2012). National suicide prevention lifeline: Enhancing mental health care for suicidal individuals and other people in crisis. Suicide Life Threat. Behav..

[32] Asarnow J.R., Baraff L.J., Berk M., Grob C.S., Devich-Navarro M., Suddath R., Piacentini J.C., Rotheram-Borus M.J., Cohen D., Tang L. (2011). An emergency department intervention for linking pediatric suicidal patients to follow-up mental health treatment. Psychiatr. Serv..

[33] Eichelberger A.H., Chouinard A.O., Jermakian J.S. (2012). Effects of booster seat laws on injury risk among children in crashes. Traffic Inj. Prev..

[34] Truong W.H., Hill B.W., Cole P.A. (2013). Automobile safety in children: A review of North American evidence and recommendations. J. Am. Acad. Orthop. Surg..

[35] King W.D., Monroe K., Applegate J., Cole-Farmer J. (2007). The impact of education, legislation and service on Alabama child passenger safety. J. Trauma.

[36] Ebel B.E., Koepsell T.D., Bennett E.E., Rivara F.P. (2003). Use of child booster seats in motor vehicles following a community campaign: A controlled trial. Jama.

[37] Korea Legislation Research Institute The Road Traffic Act in Republic of Korea 2018, Article 50 (subparagraph 5(1)). https://elaw.klri.re.kr/eng_service/lawView.do?lang=ENG&hseq=906.

[38] Du R.Y., LoPresti M.A., Garcia R.M., Lam S. (2020). Primary prevention of road traffic accident-related traumatic brain injuries in younger populations: A systematic review of helmet legislation. J. Neurosurg. Pediatr..

[39] Karkhaneh M., Rowe B.H., Saunders L.D., Voaklander D.C., Hagel B.E. (2013). Trends in head injuries associated with mandatory bicycle helmet legislation targeting children and adolescents. Accid. Anal. Prev..

[40] Kanny D., Schieber R.A., Pryor V., Kresnow M.J. (2001). Effectiveness of a state law mandating use of bicycle helmets among children: An observational evaluation. Am. J. Epidemiol..

