# Supplementary material for: National Surveillance of Injury in Children and Adolescents in the Republic of Korea: 2011–2017
Source: Int J Environ Res Public Health. 2020 Dec 7;17(23):9132. doi: 10.3390/ijerph17239132 (PMC7731276; doi:10.3390/ijerph17239132)
Supplement: Supplementary file 1 [file ijerph-17-09132-s001.zip › ijerph-999892-supplementary/supplementary_final/ijerph-supplementary_201201_plain.docx]

**Supplementary materials**

**Table S1.** ICECI version 1.2 and EDIIS code equivalents: mechanism/cause

| **Mechanism/cause** | **ICECI 1.2** | **EDIIS** | **Mode (our study)** |
| --- | --- | --- | --- |
| Fall | C2 = 1.5 | C12 (C12.0, C12.1, C12.2, C12.3, C12.4, C12.5, C12.6, C12.7, C12.8, C12.9) | Fall, slip |
| Struck by, against | C2 = 1.2 | C13 (C13.2 (C13.21, C13.22, C13.23, C13.25, C13.28, C13.29), C13.3, C13.4, C13.9) | Collision |
| Cut/ pierce | C2 = 2 (except 2.2.2) | C2 (C2.1, C2.2 (C2.20, C2.22, C2.23, C2.24), C2.4 (C2.41, C2.42, C2.43, C2.45, C2.47, C2.48), C2.8, C2.9) | Penetration |
| Overexertion | C2 = 7 | C7 | Overuse |
| Fire/burn | C2 = 4.1, C2 = 4.1.3, C2 = 4.1.1;4.1.2; 4.1.4 to 4.1.9 | C4 (C4.1(C4.11, C4.12, C4.13, C4.14, C4.15, C4.16, C4.17, C4.18, C4.19), C4.2 (C4.21, C4.22, C4.28. C4.29), C4.8, C4.9) | Thermal injury |
| Motor vehicle (MV) traffic | C2 = 1.1 AND any of the Transport module statements for the subordinate MV traffic rows. | C11 (C11.10, C11.20, C11.45, C11.61, C11.67, C11.70, C11.82, C11.84, C11.88, C11.90, C11.92, C11.98, C11.99) (and use of specific MV mechanism code: TA_COL_PATTERN) | Motor vehicle |
| Poisoning | C2 = 6 | C6 (C6.1 (C6.11, C6.12, C6.13, C6.18, C6.19), C6.2 (C6.21, C6.22, C6.23, C6.28, C6.29, C6.8, C6.9) | Substance exposure |
| Drugs | C2 = 20, 20.1, 20.3 to 20.9 | C6 (and use of drug classification code: PO_MACD C1) |  |
| Drowning/submersion | C2 = 5.2 | C5.1 | Drowning, hanging, asphyxia |
| Suffocation | C2 = 5.1; 5.3 to 5.9 | C5.2 (C5.21, C5.22, C5.23, C5.24, C5.25), C5.3, C5.8, C5.9 |  |
| Machinery | C2 = 3.2 | C3 (C3.1, C3.2, C3.8, C3.9) | Machine |
| Natural/environmental | C2 = 2.2 or 8 | C8 (C8.1, C8.2, C8.5, C8.8, C8.9) | Natural disaster |
| Other specified, classifiable | C2 = 1.3 to 1.8; 3.1; 3.8; 4.2; 4.8 | C98 (C98.1 (C98.11, C98.12, C98.13, C98.14, C98.15, C98.18, C98.19) C98.2 (C98.21, C98.22, C98.24, C98.27), C98.3, C98.4, C98.6) | Others and unknown |
| Other specified, not elsewhere classifiable | C2 = 1.9; 3.9; 4.9; 98 | C98.8 |  |
| Unspecified | C2 = 99 | C99 |  |

**Table S2.** Emergency department visits for injury by pediatric age group and year.

| **Year** | **Group 1**  **(0–4)** | **Group 2**  **(5–9)** | **Group 3**  **(10–14)** | **Group 4**  **(15–19)** | **Total visits**  **by year** |
| --- | --- | --- | --- | --- | --- |
| 2011 | 40023 | 17435 | 12335 | 14009 | 83802 |
| 2012 | 45535 | 19362 | 12554 | 14509 | 91960 |
| 2013 | 46486 | 19978 | 11837 | 13567 | 91868 |
| 2014 | 47976 | 21880 | 12415 | 14397 | 96668 |
| 2015 | 46118 | 20827 | 12108 | 14990 | 94043 |
| 2016 | 47508 | 21504 | 11866 | 14796 | 95674 |
| 2017 | 48025 | 22489 | 12459 | 14144 | 97117 |
| **Total visits by age group** | 321671 | 143475 | 85574 | 100412 | 651132 |

**Table S3.** Hospital length of stay and death of pediatric injury inpatients.

| **Outcome** | **Group 1 (0–4)**  **(*n =* 7183)** | **Group 2 (5–9)**  **(*n =* 7451)** | **Group 3 (10–14)**  **(*n =* 6358)** | **Group 4 (15–19)**  **(*n =* 9478)** | ***p* value** |
| --- | --- | --- | --- | --- | --- |
| **Length of stay,**  **median (IQR), days** | 2.8 (1.7-5.7) | 3.7 (2.0-7.0) | 4.8 (2.7-8.8) | 7.6 (3.7-14.8) | <0.001 |
| **Hospital death, No. (%)** |  |  |  |  | <0.001 |
| - No | 7108 (99.0%) | 7401 (99.3%) | 6315 (99.3%) | 9326 (98.4%) |  |
| - Yes | 75 (1.0%) | 50 (0.7%) | 43 (0.7%) | 152 (1.6%) |  |

**Table S4.** (a) The number of children aged ≥6 years who wore a safety seat belt and experienced air bag installation at the time of the car crash. (b) The number of children aged 6 years who experienced air bag inflation at the time of the car crash.

| **(a)** | **No. (%)** | | | ***p* value** |
| --- | --- | --- | --- | --- |
|  | **6–9**  **(*n =* 820)** | **10–14**  **(*n =* 623)** | **15–19**  **(*n =* 1357)** |  |
| **Safety seat belt** |  |  |  | 0.683 |
| - Yes | 358 (43.7%) | 281 (45.1%) | 574 (42.3%) |  |
| - No | 409 (49.9%) | 297 (47.7%) | 695 (51.2%) |  |
| - Unknown | 53 (6.5%) | 45 (7.2%) | 88 (6.5%) |  |
| **Air bag installation** |  |  |  | 0.068 |
| - Yes | 201 (24.5%) | 122 (19.6%) | 290 (21.4%) |  |
| - No | 153 (18.7%) | 117 (18.8%) | 224 (16.5%) |  |
| - Unknown | 466 (56.8%) | 384 (61.6%) | 843 (62.1%) |  |

| **(b)** | **No. (%)** | | | ***p* value** |
| --- | --- | --- | --- | --- |
|  | **6–9**  **(*n =* 201)** | **10–14**  **(*n =* 122)** | **15–19**  **(*n =* 290)** |  |
| **Air bag inflation** |  |  |  | 0.001 |
| - Yes | 26 (12.9%) | 21 (17.2%) | 82 (28.3%) |  |
| - No | 168 (83.6%) | 97 (79.5%) | 203 (70.0%) |  |
| - Unknown | 7 (3.5%) | 4 (3.3%) | 5 (1.7%) |  |

**Table S5.** The number of children aged under 6 years sitting on the car safety seat at the time of the car crash.

|  | **No. (%)** | | | ***p* value** |
| --- | --- | --- | --- | --- |
|  | **0–1**  **(*n =* 2422)** | **2–3**  **(*n =* 2455)** | **4–5**  **(*n =* 2021)** |  |
| **Car safety seat** |  |  |  | <0.001 |
| - Yes | 344 (46.3%) | 286 (38.4%) | 172 (28.5%) |  |
| - No | 352 (47.4%) | 386 (51.8%) | 347 (57.5%) |  |
| - Unknown | 47 (6.3%) | 73 (9.8%) | 84 (13.9%) |  |

**Table S6.** The number of adolescents wearing helmet, aged ≥10 years, at the time of motorcycle-related injury as a driver or a passenger.

|  | **No. (%)** | | ***p* value** |
| --- | --- | --- | --- |
|  | **10–14**  **(*n =* 46)** | **15–19**  **(*n =* 2237)** |  |
| **Helmet-wearing** |  |  | 0.009 |
| - Yes | 6 (13.0%) | 765 (34.2%) |  |
| - No | 36 (78.3%) | 1282 (57.3%) |  |
| - Unknown | 4 (8.7%) | 190 (8.5%) |  |

**Table S7.** The number of individuals wearing helmet by age group at the time of the bicycle-related injury .

|  | **No. (%)** | | | | ***p* value** |
| --- | --- | --- | --- | --- | --- |
|  | **Group 1 (0-4)**  **(*n =* 296)** | **Group 2 (5-9)**  **(*n =* 1724)** | **Group 3 (10-14)**  **(*n =* 2857)** | **Group 4 (15-19)**  **(*n =* 1619)** |  |
| **Helmet-wearing** |  |  |  |  | <0.001 |
| - Yes | 23 (7.8%) | 147 (8.5%) | 242 (8.5%) | 146 (9.0%) |  |
| - No | 220 (74.3%) | 1313 (76.2%) | 2343 (82.0%) | 1331 (82.2%) |  |
| - Unknown | 53 (17.9%) | 264 (15.3%) | 272 (9.5%) | 142 (8.8%) |  |
