# Supplementary figures and images for: National Surveillance of Injury in Children and Adolescents in the Republic of Korea: 2011–2017
Source: Int J Environ Res Public Health. 2020 Dec 7;17(23):9132. doi: 10.3390/ijerph17239132 (PMC7731276; doi:10.3390/ijerph17239132)

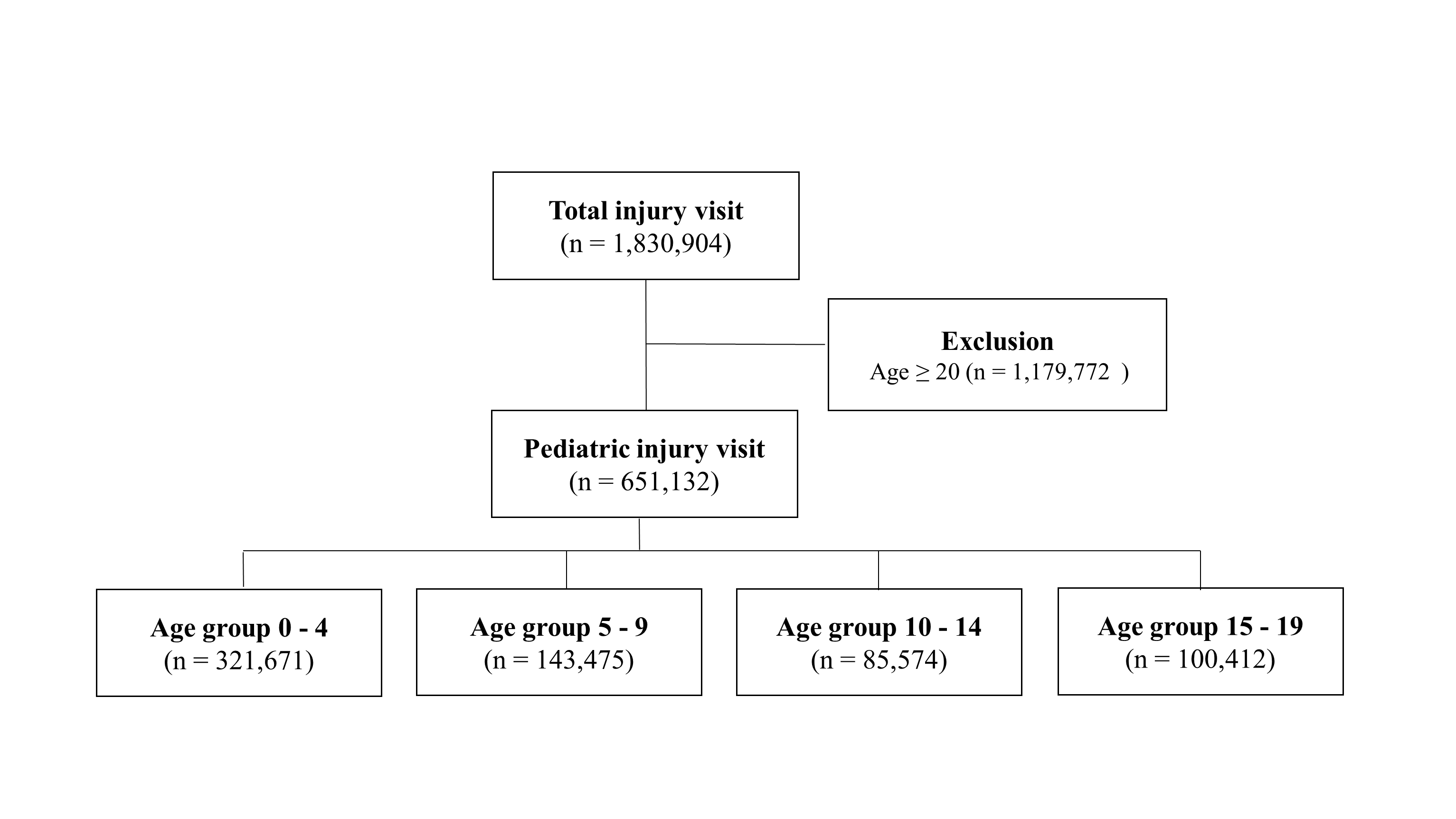

Supplement: Supplementary file 1 [file ijerph-17-09132-s001.zip › ijerph-999892-supplementary/supplementary_final/figure S1.tif]

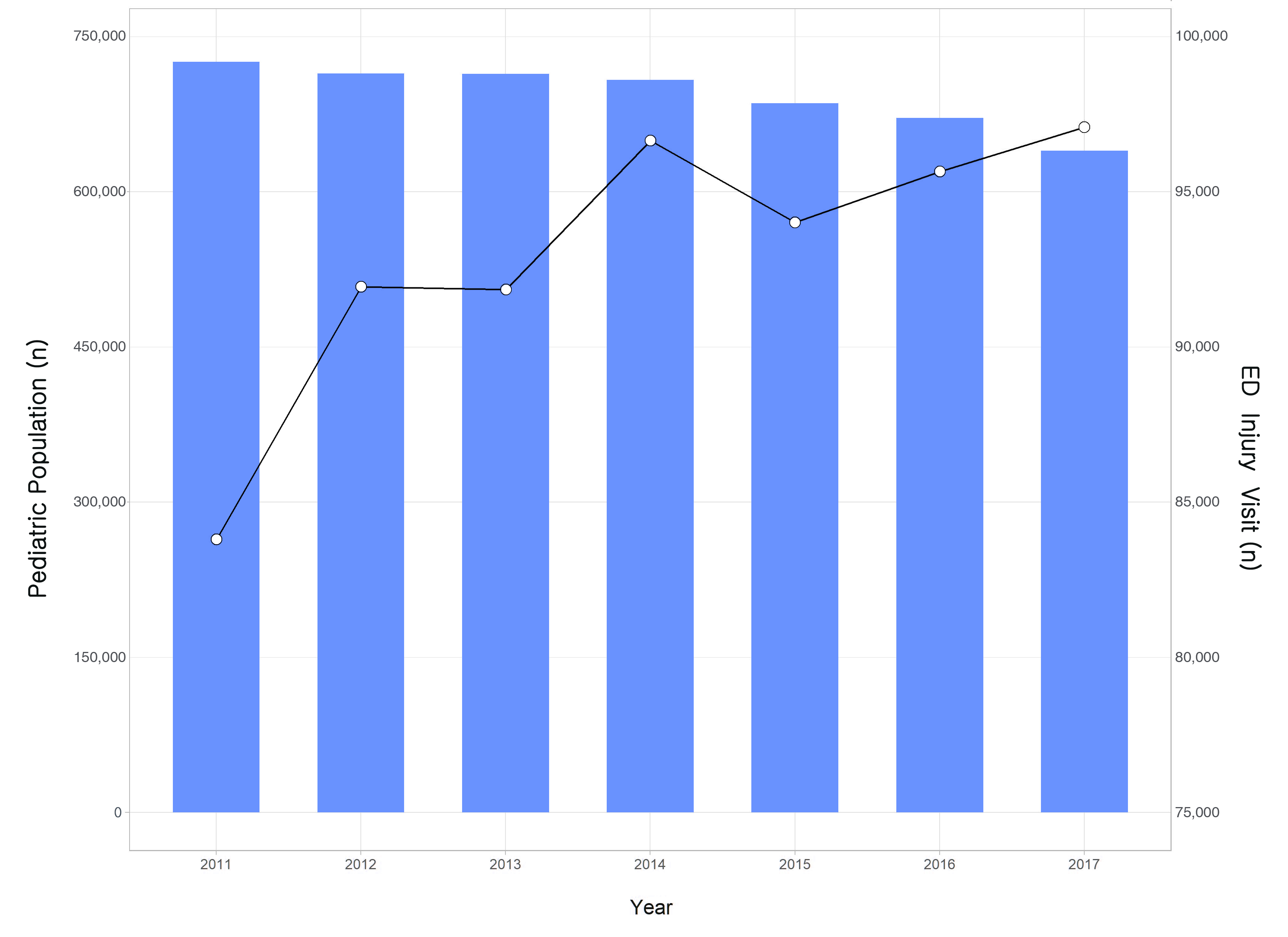

Supplement: Supplementary file 1 [file ijerph-17-09132-s001.zip › ijerph-999892-supplementary/supplementary_final/FigureS2.jpg]
